# Knowledge and Practice of Neonatal Pain Management and Associated Factors among Health Care Providers in Neonatal Intensive Care Units of Public Hospitals in North Shoa Zone, Amhara Regional State, Ethiopia, 2023

**DOI:** 10.1155/2024/9997231

**Published:** 2024-07-22

**Authors:** Abraraw Admasu Jember, Eyosiyas Yeshialem Asefa, Abdurahman Mohammed Ahmed, Addis Yeshitila Kidane

**Affiliations:** ^1^ School of Medicine Asrat Woldeyes Health Science Campus Debre Berhan University, Debre Berhan, Ethiopia; ^2^ School of Public Health Asrat Woldeyes Health Science Campus Debre Berhan University, Debre Berhan, Ethiopia

## Abstract

**Background:**

Neonatal pain has been underrecognized and undertreated in many settings with the youngest children and neonates suffering the greatest consequences. Despite recent advancements in the assessment and therapy of children's pain, a knowledge-to-practice gap still exists especially in developing nations including our country Ethiopia.

**Objectives:**

To assess knowledge, practice, and associated factors towards neonatal pain management among health care professionals working at neonatal intensive care units of public hospitals in North Shoa Zone, Amhara regional state, Ethiopia.

**Method:**

Facility-based cross-sectional study design was conducted among health care professionals working at NICU in all public hospitals in North Shoa Zone, from May 9, 2023, to May 23, 2023. Data were collected using a self-administered questionnaire from 123 health care professionals working at NICUs in eleven public hospitals. The collected data were checked and entered into EPI data version 3.1 and then exported to SPSS version 25 for further descriptive and logistic regression analysis. Data are summarized using descriptive statistics and presented using narrations, tables, and graphs.

**Result:**

The study reveals that 48% of health care professionals possessed adequate knowledge, while only 5.7% demonstrated good practices in neonatal pain management. Factors significantly associated with better neonatal pain management practices included having good knowledge of neonatal pain management (AOR = 3.36, 95% CI [1.19–9.49]), a higher educational level (AOR = 4.48, 95% CI [1.62–13.88]), and the availability of a pain scale assessment tool in the neonatal unit (AOR = 3.91, 95% CI [1.50–10.20]). Moreover, the type of profession, specifically being a nurse (AOR = 0.23, 95% CI [0.08–0.66]), was significantly associated with knowledge of neonatal pain management.

**Conclusion:**

Health care professionals' knowledge and practice regarding neonatal pain management are insufficient. Multisectoral collaboration is essential to enhance their knowledge and skills and improve the availability of pain scale assessment tools and supportive materials.

## 1. Introduction

The International Association for the Study of Pain (IASP) definition includes the clear connection between pain and tissue damage, the existence of dualistic thinking about body and mind, and the lack of stress. It resolved between the primacy of self-assessment and the privileging of the observer's perspective [1]. In 2020, the IASP revised its definition of pain, an unpleasant sensory and emotional experience associated with actual or potential tissue damage [2].

Newborns experience acute pain during various medical procedures. Evidence demonstrates that pain control in the neonatal period is beneficial because it improves physiological, behavioral, and hormonal outcomes [3]. Newborns undergo an average of 6.6 invasive procedures per day [4].

Historically, pain in children has been underrecognized and poorly treated by health care professionals, and although advances in assessment and treatment have been made in recent years, there is still approximately a gap between knowledge and practice [5]. Age-appropriate pain assessment should be available, using validated assessment tools [6]. Preventing pain in infants should be the goal of all caregivers because repeated exposure to pain can have serious consequences [7].

Pain assessment in nonverbal children and infants can be a very difficult task in an already subjective process [3]. Pain assessment and management tools should be standardized across trusts in youth care areas [6]. There are several validated scoring systems for assessing pain in neonates; however, there is no standardized or universal approach to pain management [3]. Assessing pain in infants is notoriously difficult due to their physical, cognitive, and behavioral development. The use of a pain rating scale ensures consistency between nurses and other clinicians and provides an accurate measure of the presence of pain, tension, or discomfort [7]. These scales not only quantify pain but can also provide an accurate description of the impact of nonpharmacological and pharmacological management interventions on neonatal pain [8].

Multiprofessional education is highly valued and seen as having the potential to enhance the knowledge of nurses and physicians. A more reflective and collaborative practice was developed following this realization [8]. Clinical staff should be aware of available pain management strategies, including nonpharmacological strategies, medication dosages, and appropriate regimens for pain control in the hospital setting [6].

Pain prevention and management guidelines should include psychological, physical, and pharmacological strategies for acute and chronic pain. Health care guidelines and practice guidelines should incorporate strategies and approaches in health care settings and home use [9].

Despite strong evidence and recommendations for pain management in national and international guidelines and organizations, knowledge has not yet been translated into consistent standard care across settings. Diverse mothers, newborns, and children, where painful neonatal procedures take place [8], most pain is preventable or at least treatable. There are many things that primary care physicians, pediatricians, and other allied health care professionals can do to reduce or eliminate pain. Pain can be effectively managed using a wide range of pharmacological, physical, and psychological approaches [10].

Neonates at the greatest risk of neurodevelopmental impairment as a result of preterm birth (the smallest and sickest) are also those most likely to be exposed to the greatest number of painful stimuli in the NICU [7]. Early exposure to painful procedures can negatively impact neurodevelopment, a greater risk of developing noncommunicable diseases (diabetes and hypertension), and other health conditions later in life [11].

## 2. Methods and Materials

### 2.1. Study Area

The study was conducted in North Shoa zone public hospital, Amhara regional state, Ethiopia. Debre Berhan is the capital city of the zone located about 120 kilometers from Addis Ababa. In the North Shoa zone, there are one comprehensive, one teaching, two general hospitals and 7 district hospitals that have neonatal care units. In these hospitals, neonatal care is given by pediatricians, general practitioners, nurses, and other health care professionals. Currently, 135 health care providers are working in the neonatal care unit.

We took study participants from 11 hospitals, namely, Hakim Gizaw Teaching Hospital, Debre Berhan Specialized Hospital, Enat Hospital, Mehalmeda Hospital, Molale Hospital, Deneba Hospital, Ataye Hospital, Shoarobit Hospital, Debresina Hospital, Arerti Hospital, and Mida Hospital.

### 2.2. Study Design and Period

Institutional-based cross-sectional study design was employed from May 9, 2023, to May 23, 2023.

### 2.3. Source Population

All health professionals working in the neonatal intensive care unit of North Shoa zone public hospitals were the source of population.

### 2.4. Study Population

The study population was health professionals working in neonatal intensive care units in North Shoa zone public hospitals.

### 2.5. Inclusion and Exclusion Criteria

#### 2.5.1. Inclusion Criteria

Health professionals must have a minimum of six months of work experience in the neonatal intensive care unit (NICU).

#### 2.5.2. Exclusion Criteria

Individuals meeting the inclusion criteria but absent, on maternity leave, or on annual leave during the data collection, and those with less than six months of work experience were excluded from the study.

### 2.6. Sample Size Determination

We determined the sample size utilizing Epi Info 7.2.6 software, assuming a 51% prevalence of insufficient knowledge about pain evaluation based on a study conducted in Turkey [12], with a 5% margin of error and a 95% confidence interval. The calculated sample size was 384. However, considering that there are only 135 health professionals currently working at the neonatal intensive care unit (NICU) in the study area, we decided to include all of them in our study.

### 2.7. Sampling Procedure

We included all 135 health care providers working in the NICU of 11 North Shoa public health hospitals, specifically Hakim Gizaw Teaching Hospital [13], Debre Berhan Specialized Hospital (35), Enat Hospital [12], Mehalmeda Hospital [12], Molale Hospital [6] Deneba Hospital [10], Ataye Hospital [8], Shoarobit Hospital [12], Debresina Hospital [8], Arerti Hospital [9], and Mida Hospital [7].

## 3. Data Collection Methods

Data were collected using a self-administered questionnaire adapted from previous research [14, 15]. The developed tool in the English language comprised four sections, encompassing sociodemographic factors, knowledge, practices, and organizational factors. The data were gathered from 123 health care providers stationed at 11 public hospitals with neonatal intensive care units.

### 3.1. Study Variables

#### 3.1.1. Dependent Variables

Knowledge and practice are there in neonatal pain management.

#### 3.1.2. Independent Variables

Sociodemographic factors included age, sex, religion, marital status, level of education, and work experience.

Organizational factors included presence of protocol and guideline, presence of analgesics, presence of standardized tool for neonatal pain assessment training, presence of assessment tool, presence of guidelines, presence of analgesics, and provision of training.

### 3.2. Operational Definitions

Adequate knowledge included those health professionals who scored equal to or greater than 80% of the knowledge questions correctly.

Inadequate knowledge included those professionals who scored below 80% on the knowledge item.

Good practice included those health professionals who scored equal to or greater than 80% of practice questions.

Poor practice included those professionals who scored below the 80% for practice-related questions.

Health care professionals included nurses, general practitioners, pediatricians, and child health specialists.

Neonatal pain management refers to assessing the degree of pain in neonates and treating it with nonpharmacological and pharmacological measures as well as applying preventive measures.

Junior health care professionals included those people with an experience level of less than 4 years.

Senior health care professionals included those people with an experience of greater than 4 years.

### 3.3. Data Process and Analysis

Data were entered into Epi data 3.1 and checked for completeness and consistency. Subsequently, the information was transferred to SPSS version 25 software for subsequent analysis. The normality of data distribution was also checked, and data adjustments were made when needed.

Analytical methods were used to identify factors that predict health professionals' knowledge and practice of neonatal pain management. Therefore, the data were fitted to a logistic regression model. Predictor variables were identified in two steps. First, bivariate analysis was performed to identify candidates of multivariable logistic regression analysis and then the identified candidates were included in multivariate analysis to control for confounding factors. The existence and magnitude of associations were established through odds ratios, 95% confidence intervals (CI), and the corresponding *p* values. Ultimately, the findings were summarized and presented through statements, tables, and graphs, utilizing adjusted odds ratios and their respective 95% CIs.

### 3.4. Data Quality Control

The instrument underwent a pretest on a 5% sample in hospitals outside of the study area. After the pretest, essential modifications were made to improve the tool's efficiency before initiating the actual data collection process. Thorough training was conducted for both the data collectors and supervisors. Supervisors conducted daily reviews and checks of the questionnaires to ensure their completeness and relevance. The tools' reliability and convergent validity were also evaluated.

### 3.5. Ethical Considerations

Ethical approval was obtained from Debre Berhan University, Asrat Woldeyes Health Sciences Facility, and the Institutional Ethics Review Board (IRB-179). Official authorization and support documents are written for the respective medical and administrative facilities. Informed consent was obtained from each study participant. Before data collection, study participants were informed about the purpose of the study and their right to refuse participation. Additionally, study participants were informed that the information obtained would be kept confidential throughout this study. For confidentiality purposes, the names of the participants were not included in the questionnaire. The collected data were kept confidential and used only for the study. This study was conducted following the Declaration of Helsinki.

## 4. Results

### 4.1. Sociodemographic Characteristics of Health Care Professionals

A total of 131 health care professionals participated in this study yielding a response rate of 123 (94%). The mean age of the participants was 30.91 years with a minimum of 24 and a maximum of 43 and ± SD3.785. Additionally, 63/123 (51.8%) were nurses, and 60/123 (48.2%) were medical doctors. Regarding professional experience, junior level accounts for 47.2% and seniors account for 52.8% (see Table 1).

### 4.2. Factors Associated with Knowledge of Health Care Professionals towards Neonatal Pain Management

Among study participants 42.3% of health professionals responded that there was no clear guideline/protocol for neonatal pain management, 52.8 had neonatal pain scale assessment tools in the unit, and 47.2% had pain management in-service training.

Anti-pain was available in 82.9% of neonatal care units and 34.45% of them had palliative care training (see Table 2).

### 4.3. Knowledge of Health Care Professionals on Neonatal Pain Management

**Figure 1:**
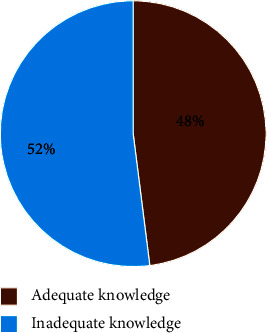
Knowledge of health care professionals on neonatal pain management among health care providers working at NICU of North Shoa zone public hospitals, Amhara regional state, Ethiopia, 2023GC.

Accordingly, in this study, 59/123 (48%) of the study participants had adequate knowledge of neonatal pain management, while others had inadequate knowledge (see Figure 1).

Regarding knowledge assessment tools, health care professionals were more agreed on the term newborn feel pain compared to preterm which was 94.3% and 92.6%, respectively, and 86.7% agreed that the pain assessment scale is important to practice. Only 65% of them agreed that pain in neonates harmed their development (see Tables 2 and 3).

### 4.4. Practice of Health Care Professionals on Neonatal Pain Management

**Figure 2:**
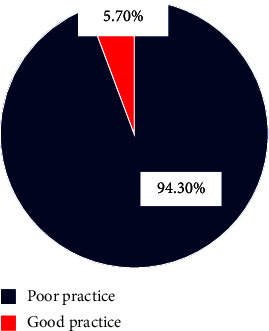
Practice of health care professionals on neonatal pain management among health care providers working at NICU of North Shoa zone public hospitals, Amhara regional state, Ethiopia, 2023GC.

According to this study, 7/123 (5.7%) of the study participants had good practice concerning neonatal pain management, while 116/123 (94.3%) of them had poor practice (see Figure 2).

Regarding the practice of assessment tools, only 7.3% always practiced assessing pain through facial expressions and 41.46% practiced it occasionally. About the scale rating tool, 11.38% of them practiced always and 26% practiced a few times.

Regarding pain control practices, only 13.8% practiced breastfeeding to relieve pain and 9.7% of them never practiced it, and 34.14 % of them never used sucrose/glucoseto relieve pain, while 30.4% health care providers have never prescribed opioids when needed for pain relief (see Table 1).

### 4.5. Factors Associated with Knowledge of Health Care Professionals on Neonatal Pain Management

Each outcome variable is tested for the presence of association using chi-square, and those who had association and *p* value less than (*p* < 0.25) bivariate logistic regression were selected as candidates for multiple regression to determine the predictor variables of neonatal pain management of health care professionals.

Among those predictors, the type of profession, presence of guidelines, pain scale availability, and pain education at higher education were associated with knowledge of health care professionals towards neonatal pain management in multivariate logistic regression analysis. Finally, model fitness was tested by the Hosmer and Lemeshow tests and fit for it.

Accordingly, the odds of having adequate knowledge of neonatal pain management among health care professionals who had neonatal pain management guidelines in the neonatal unit is 3.365 times higher than compared to those who had no guideline (AOR 3.362 (1.190–9.496)), the odds of having adequate knowledge towards neonatal pain management among health care professionals who had adequate education to manage neonatal pain is 4.478 times higher than compared to who had no adequate education (AOR = 4.478, CI (1.624–13.884)), and the odds of having sufficient knowledge of neonatal pain management among health care professionals who had a pain scale assessment tool in the neonatal unit are 3.913 more likely compared to those who had no pain scale tool (AOR 3.913(1.500–10.204). The study also revealed that nurses were 76.7% less likely to have complete knowledge of pain management in newborns than doctors (AOR = 0.233(0.082–0.664) (see Table 4).

### 4.6. Factors Associated with the Practice of Health Care Professionals on Neonatal Pain Management

Due to low health care professional neonatal pain management practices, 7/123 (5.7%), not all variables met the chi-square assumption; therefore, each variable was examined of the associations present in the Fisher exact test, and data are adapted to this type of test.

Finally, continuing education, availability of NICU pain assessment tools, and palliative care were significantly associated with health care professionals' practice of neonatal pain management (see Table 5).

## 5. Discussion

### 5.1. Knowledge of Health Care Professionals on Neonatal Pain Management

In this investigation, health care professionals demonstrated a comprehensive understanding of pain management in newborns, registering at 48% with a confidence interval of (32–56). This finding was found to be consistent with a study conducted in Turkey, where 51% of participants reported insufficient knowledge regarding pain assessment and management [12].

On the other hand, a descriptive cross-sectional study conducted in Kigali, focusing on neonatal pain management among nurses and midwives in two hospitals, revealed that a majority (74.2%) exhibited limited knowledge about pain in newborns and its management [9] which is found to be above our finding. This discrepancy might be due to sociodemographic differences among the study participants and other study setting-related issues.

The outcome of our study indicates a lower percentage compared to studies conducted in Ethiopia, specifically in Addis Ababa, West Oromia, and Mekele, where 68.7%, 62.6%, and 58.6% of nurses demonstrated sufficient knowledge of pain management, respectively [13, 16, 17]. This disparity could be attributed to the fact that the aforementioned studies exclusively focused on nurses working in urban hospitals, whereas our research included doctors, midwives, and nurses working in neonatal intensive care units (NICUs) at primary hospitals.

In the evaluation of knowledge assessment tools, health care professionals demonstrated a higher consensus on understanding pain in full-term infants compared to preterm infants, with agreement percentages of 94.3% and 92%, respectively. There is an observed discrepancy in the management of newborns compared to full-term infants, and only 65% of health care professionals are aware of the detrimental effects of painful procedures on their development. Consequently, pain tends to be overlooked in this particular population. These findings align with studies conducted in Ethiopia, specifically in Addis Ababa and western Oromia [13, 16].

### 5.2. Knowledge Associated Factors of Health Care Professionals on Neonatal Pain Management

The findings of this study indicate that factors such as sufficient education, the presence of guidelines within the facility, the availability of pain scale assessment tools in the unit, and the specific profession of health care professionals are linked to their knowledge of neonatal pain management.

Accordingly, the odds of having adequate knowledge of neonatal pain management among health care professionals who had neonatal pain management guidelines in the neonatal unit are 3.37 times higher compared to those who had no guideline AOR 3.362 (CI: 1.190–9.496). This is consistent with a study performed in Turkey where the availability of guidelines at the NICU was directly related to the knowledge of health care professionals on pain management [12]. This linkage may be justified by the fact that guidelines serve as structured reference materials, providing standardized information and protocols. Health care professionals are likely to enhance their understanding and adherence to best practices when clear guidelines are accessible, contributing to improved knowledge and competency in neonatal pain management.

The observed increase in the odds of having good neonatal pain management knowledge among health care professionals who had adequate education to manage neonatal pain (AOR = 4.478, CI: 1.624–13.884) can be justified by the pivotal role of education in shaping health care professionals' understanding and competence in neonatal care. Adequate education likely provides professionals with a solid foundation of knowledge and skills necessary for effective pain management in neonates. This finding aligns with a study conducted in Addis Ababa and West Oromia, where a positive association was identified between having adequate knowledge and better neonatal pain management knowledge [13]. The consistent pattern across studies underscores the importance of education as a key determinant in promoting proficiency and expertise in neonatal pain management among health care professionals.

The observed odds, where nurses are 76.7% less likely to have adequate knowledge of neonatal pain management compared to medical doctors (AOR = 0.233, CI: 0.082–0.664), may be justified by the variations in educational backgrounds, training, and specialization between the two professions. Medical doctors typically undergo extensive medical education and training, which may encompass a more comprehensive understanding of neonatal care, including pain management. Additionally, the nature of their roles and responsibilities in patient care may expose doctors to a wider range of medical knowledge and experiences compared to nurses. This difference in educational and professional factors could contribute to the significant contrast in the likelihood of having adequate knowledge between the two professions.

The substantial increase in the odds of having adequate knowledge about pain management in newborns, with a 3,913 times higher likelihood, among health care professionals who had a pain assessment tool in the neonatal unit compared to those without such tools (AOR 3.913, CI: 1.500–10.204), can be justified by the instrumental role that these tools play. Pain assessment tools provide a systematic and standardized approach to evaluating pain in neonates, offering health care professionals a tangible and structured means to identify and address pain. The availability of such tools likely enhances professionals' familiarity with effective pain management strategies, contributing to the significant difference in knowledge levels observed between those with and without access to pain assessment tools.

### 5.3. Practice of Health Care Professionals on Neonatal Pain Management

In this study, about 5.7% of health professionals had good neonatal management practice with CI (1.6–10.6) which is lower than a study performed in Rwanda and West Ormia (15.2%) and 32.2% of health providers have good neonatal pain management practice, respectively [9, 18]. The possible justification for this discrepancy could be due to the variation in study settings and the use of modified tools from these studies.

Regarding pain management practices, only 13.8% practiced breastfeeding to relieve pain, 9.7% of them never practiced, and sucrose/glucose was never practiced to relieve pain in 34.14% of health care providers as well they never prescribed opioids when necessary to relieve pain in 30.4%, indicating that there is a low practice of both nonmedication and medication use to relieve neonatal pain which is comparable to a study performed in West Oromia [18]. The observed patterns may be indicative of a broader issue, such as a lack of awareness, training, or institutional protocols, emphasizing the need for targeted interventions and education to enhance neonatal pain management practices among health care providers.

In this study, having palliative care and in-service pain management training were significantly associated with good neonatal pain management practice. This is found to be consistent with a study performed in West Oromia where nurses with adequate knowledge were more likely to adopt good neonatal pain management practices than those without adequate knowledge [13] and with a study conducted in Addis Ababa that showed nurses applying neonatal pain management policies were more likely to implement good practices in neonatal pain management [16]. These consistent findings across studies underscore the crucial role of education and training in shaping health care professionals' practices in neonatal pain management.

## 6. Conclusion

In this study, health care professionals demonstrated a 48% understanding of neonatal pain management, lower than studies in Turkey and Rwanda. Factors influencing knowledge included education, the presence of guidelines, and the specific profession. Nurses were 76.7% less likely to have adequate knowledge compared to medical doctors, emphasizing the need for targeted interventions.

The study found associations between having guidelines and education with better knowledge. The odds of adequate knowledge were 3.37 times higher among those with guidelines, aligning with findings in Turkey. Additionally, the low prevalence of good neonatal pain management practices suggests a need for increased awareness and training. The positive impact of palliative care and in-service pain management training underscores the critical role of education in shaping health care professionals' practices.

## 7. Recommendations

For the Ministry of Health,

1. Providing related training for professionals and improving the distribution of supporting guidelines and tools

For Debre Berhan University,

1. Developing supportive guidelines and tools and providing training regularly
2. Doing regular supervision and mentorship to see effectiveness

For Zonal Health Bureau,

1. Facilitating and providing regular training and ensuring that professionals adhere to developed guidelines
2. Development of consistent guidelines for neonatal pain management by the MOH and the concerned body

For researchers,

1. The study was done with the limitation of a small sample size, so we recommend at large scale by using better study designs

## 8. Strengths and Limitations of the Study

### 8.1. Limitations of the Study

The practice was assessed by a self-administered questionnaire; it would be better if it was supported with qualitative data.

Limited research parallel to our study makes it difficult to discuss in detail.

### 8.2. Strengths of the Study

It is a pioneer study in neonatal pain management involving health care professionals as a team in our country.

## Figures and Tables

**Table 1:** Sociodemographic characteristics of health care professionals towards neonatal pain management among health care providers working at NICU of North Shoa zone public hospitals, Amhara regional state, Ethiopia, 2023GC.

Variables	Categories	Number	Percent
Sex	Male	78	63.4
	Female	45	36.6

Marital status	Single	57	46.3
	Married	65	52.8
	Widowed	1	0.8

Religion	Orthodox	115	93.1
	Others	8	6.9

Professional	Diploma nurse	7	5.7
	BSc nurse	42	34.7
	NICU nurse	14	11.4
	General practitioner	54	43.9
	Pediatrics and child health specialty	6	4.9

Work experience	Less than 4 years	58	47.2
	Greater than 4 years	65	52.8

**Table 2:** Factors associated with knowledge and practice of health care professionals towards neonatal pain management among health care providers working at NICU of North Shoa zone public hospitals, Amhara regional state, Ethiopia, 2023GC.

Variables	Yes		No	
	No	%	No	%
Adequate education on pain management of neonates	71	57.7	52	42.3
Pain management training received at higher education previously	47	38.2	76	61.8
Pain management in-service training received previously	65	52.8	58	47.2
Pediatric palliative care training received previously	46	37.4	77	62.6
Presence of guide for neonatal pain management in institutions	71	57.7	52	42.3
Availability of scale assessment tools for neonatal pain in the unit	65	52.8	58	47.2
Available antipain in the neonatal unit	102	82.9	21	17.1

**Table 3:** Knowledge characteristics of neonatal pain management among health care providers working at NICU of North Shoa zone public hospitals, Amhara regional state, Ethiopia, 2023GC.

Variables	Agree		Disagree		Unsure	
	No	%	No	%	No	%
(1) Preterm newborns feel pain	114	92.6	8	6.5	1	0.8
(2) Full-term newborns feel pain	116	94.3	6	4.8	1	0.8
(3) Pain can affect newborns' heart rate, respiratory rate, temperature, blood pressure, oxygen saturation, and intracranial pressure	116	94.3	5	4	2	1.6
(4) Pain can affect newborns' facial expressions, limb movements, and crying	109	88.6	13	10.6	1	0.8
(5) Light and noise may affect newborns' reactions to pain	96	78.04	19	15.44	8	6.5
(6) Newborns' pain is not recognized by professionals	52	42.27	77	62.60	4	3.25
(7) Newborns' pain is not considered by researchers	53	43.08	64	52.03	6	4.8
(8) Newborns react to pain in a particular way	84		32		6	
(9) Pain is considered as one of the vital signs in newborns	100	81.3	11	8.9	2	1.6
(10) Pain assessment in newborns must be systematized	109	88.61	11	8.94	3	2.43
(11) Pain assessment should be part of the nursing/caregiver prescription	113	91.86	10	8.13	0	0
(12) Newborns require painkillers due to the maturity of the nervous system to feel pain	86	69.9	28	22.76	9	7.3
(13) Neonatal pain can be assessed without the use of scales	41	33.3	68	55.28	14	11.38
(14) The use of scales for pain assessment is important to the practice	106	86.17	12	9.7	5	4
(15) It is important to record pain on the newborns' chart	107	86.99	14	11.38	2	1.62
(16) Recording pain assessment is a prerequisite to control	108	87.8	13	10.6	2	1.6
(17) Have sufficient knowledge to assess pain in newborns	68	55.28	47	38.21	8	6.5
(18) Pain management in newborns depends on its assessment	109	88.61	12	9.75	2	1.62
(19) Recording newborns' pain assessment will result in relief	102	8.29	18	14.63	3	2.43
(20) Repeat painful procedures may have harmful effects on their development	80	65	23	18.69	20	16.26

**Table 4:** Factors associated with knowledge of health care professionals towards neonatal pain management among health care providers working in NICU of North Shoa zone public hospitals, Amhara regional state, Ethiopia, 2023GC.

Variables		Knowledge category		(95% CI)		
				COR	AOR	*P* value
		Adequate	In adequate			
Adequate education to manage neonatal pain	Yes	46	25	5.520 (2.494–12.217)	4.478 (1.624–13.884)	0.004
	No	13	39	1	1	1

Palliative care training	Yes	30	16	1.448 (2.372–6.649)	0.941 (0.256–3.453)	0.927
	No	29	48	1	1	1

Presence of neonatal pain management guideline	Yes	47	24	6.528 (2.901–14.691)	3.362 (1.190–9.496)	0.022
	No	12	40	1	1	1

In-service pain management training	Yes	43	22	5.131 (2.372–11.100)	1.645 (0.585–4.619)	0.345
	No	16	42	1	1	1

Type of profession	Nurse	24	39	0.440 (0.213–0.905)	0.233 (0.082–0.664)	0.006
	Medical doctor	35	25	1	1	1

Pain scale tool in the unit	Yes	44	21	6.006 (2.741–13.164)	3.913 (1.500–10.204)	0.005
	No	15	43	1	1	1

Training at higher education	Yes	34	13	5.335 (2.401–11.855)	2.700 (0.684–10.649)	0.156
	No	25	51	1	1	1

**Table 5:** Factors associated with practice status of health care professionals towards neonatal pain management among health care providers working at NICU of North Shoa zone public hospitals, Amhara regional state, Ethiopia, 2023GC.

Variables		Practice status category		Fisher exact test (*p* value <0.05)
		Good	Poor	
Palliative care training	Yes	6	40	0.011
	No	1	76	

In-service pain management training	Yes	7	58	0.014
	No	0	58	

Pain scale assessment tools	Yes	5	60	0.012
	No	2	56	

## Data Availability

The data used to support the findings of this study are available from the corresponding author upon reasonable request.

## Abbreviations

AOR: Adjusted odds ratio
CI: Confidence interval
COR: Crude odds ratio
DC: Data collector
IASP: International Association for the Study of Pain
KAP: Knowledge, attitude, and practice
MOH-E: Ministry of Health-Ethiopia
NB: Newborn
NICU: Neonatal intensive care units
PI: Principal investigator
SPSS: Statistical Package for Social Sciences.
